# Immune checkpoint molecule expression is altered in the skin and peripheral blood in vasculitis

**DOI:** 10.1038/s41598-021-99558-5

**Published:** 2021-10-08

**Authors:** Chie Miyabe, Yupeng Dong, Takaharu Ikeda, Kazuo Takahashi, Yoshishige Miyabe, Tamihiro Kawakami

**Affiliations:** 1 Division of Dermatology, Tohoku Medical and Pharmaceutical University, Sendai, Japan; 2 Department of Dermatology, Tokyo Women’s Medical University, Tokyo, Japan; 3 Department of Cell Biology, Institute for Advanced Medical Sciences, Graduate School of Medicine, Nippon Medical School, Tokyo, Japan

**Keywords:** Immunology, Biomarkers, Diseases, Medical research, Rheumatology

## Abstract

Dysfunction of immunoinhibitory signals and persistent T cell activation reportedly play important roles in the development of vasculitis. The skin is one of the most accessible organs, and it is suitable for the characterization of immune cell signatures. However, the inhibitory checkpoint molecules in the skin and their relevance to vasculitis have not been studied. Here, we investigated the profile of immune checkpoint molecules in the skin and peripheral blood of patients with vasculitis and healthy donors. We found that some of the inhibitory checkpoint molecules, including programmed cell death 1 receptor (PD-1), were elevated in T-cells in the blood of patients with systemic and cutaneous vasculitis. In addition, programmed death-ligand 1 (PD-L1) expression was elevated in the skin of patients with cutaneous vasculitis. Histologically, PD-L1 was highly expressed in the vessels in the skin along with CD4^+^ and CD8^+^ T-cell infiltration in patients with cutaneous vasculitis. Notably, plasma soluble PD-L1 levels were increased, and these correlated with C-reactive protein in patients with systemic vasculitis. Our findings suggest that inhibitory checkpoint molecules might be differentially modulated in the skin and peripheral blood of patients with vasculitis, and that the alteration of the PD-L1/PD-1 axis may be associated with the regulation of T-cell activation in vasculitis.

## Introduction

Vasculitis is characterized by the inflammation of blood vessels, resulting in ischemic tissue injury. Vasculitis can affect any organ, depending on the size of the vessels involved, including skin^1^. Cutaneous vasculitis usually affects the small- or medium-sized vessels in the skin. Moreover, it includes a wide spectrum of conditions ranging from skin-limited lesions to systemic disease^2^. Most cases of isolated cutaneous vasculitis are self-limited and resolve spontaneously over a period of 3 to 4 weeks^3^. In cases of severe or recurring vasculitis, systemic therapy is required. However, the pathogenesis of vasculitis is still largely unknown, and appropriate treatment for intractable cases has not been fully established.

Recent experimental and genetic studies have demonstrated the pathogenic role of immune checkpoint molecules in vasculitis^4^. Inhibitory immune checkpoints provide negative signals to control T-cell activation and thus prevent inflammation-associated tissue damage. The most broadly studied checkpoint molecules are programmed cell death 1 (PD-1) and cytotoxic T lymphocyte-associated molecule-4 (CTLA-4). PD-1 expressed on the T-cells binds with its ligands programmed death-ligand 1 (PD-L1) or PD-L2. The PD-L1/PD-1 interaction reduces cytokine synthesis and glucose metabolism as well as blocks T-cell proliferation and survival^5^. Thus, PD-L1/PD-1 blockades became a new therapeutic strategy for reactivating the T-cell response; this had positive outcomes for patients with advanced solid tumors^6^. Furthermore, novel inhibitory pathways are under investigation, including drugs blocking lymphocyte activation gene-3 (LAG-3), T-cell immunoglobulin and mucin-domain containing-3 (TIM-3), T-cell immunoglobulin and ITIM domain (TIGIT), and V-domain Ig suppressor of T cell activation (VISTA)^7^.

The use of checkpoint inhibitors results in an excessive T-cell immune response that can cause severe immune-related adverse events (irAEs), affecting multiple organs, such as in the case of vasculitis^8^. To date, there have been more than 60 reported cases of patients developing vasculitis after receiving immune checkpoint therapy^4,9–16^. In these studies, large, medium, and small vessels were affected and even induced a flare of underlying vasculitis^12^. Usually, symptoms of vasculitis resolve after either discontinuing immune checkpoint therapy and/or administering glucocorticoids. These findings suggest that immunoinhibitory signal dysfunction and persistent T-cell activation both play important roles in the development of vasculitis.

Genetic analyses indicate that gene polymorphisms of immune checkpoint molecules may affect an individual’s susceptibility to vasculitis^17,18^. Furthermore, gene signatures associated with CD8^+^ T-cell exhaustion predicted favorable prognosis in anti-neutrophil cytoplasmic antibody (ANCA)-associated vasculitis (AAV)^19^. There have been contrasting reports on the levels of PD-1-expression in giant cell arteritis (GCA) and granulomatosis with polyangiitis (GPA). The frequency of PD-1^+^ T-cells in the peripheral blood of GPA patients increased compared with that in healthy subjects^20^. Conversely, the frequency of PD-1^+^ T-cells in the peripheral blood of GCA patients was decreased; however, the expression of PD-1 increased in the affected arteries^21^. It is still unclear how the PD-L1/PD-1 axis contributes to the development of vasculitis in the peripheral blood and inflamed skin.

This study aims to gain insights into whether immune checkpoint molecules are associated with the progression or resolution of vascular inflammation by investigating immune checkpoint molecule signatures in the skin and peripheral blood of patients with vasculitis. We demonstrate that the profiles of immune checkpoint molecules were altered the skin and peripheral blood in patients with systemic and cutaneous vasculitis. Specifically, inflamed skin highly expressed PD-L1 compared to healthy skin. Moreover, circulating T-cells in the peripheral blood preferentially expressed inhibitory immune checkpoint receptors, such as PD-1 in systemic and cutaneous vasculitis, and plasma soluble PD-L1 (sPD-L1) levels were elevated in systemic vasculitis. Presumably, the inflammatory microenvironment in vasculitis might alter PD-L1/PD-1 expression to regulate vascular inflammation.

## Results

### Characteristics of vasculitis patients and healthy donors

A total of 19 patients with vasculitis (cutaneous vasculitis: n = 14, systemic vasculitis: n = 5) and 16 healthy donors (HD) were recruited for this study. All patients were newly diagnosed without prior treatment, including immunosuppressive agents. The characteristics of patients with vasculitis and HD are depicted in Table 1. There were no significant differences between patients with vasculitis and HD in terms of age and sex.

**Table 1:** Characteristics of the patients with vasculitis and the healthy donors.

	Healthy donors	Vasculitis cases	*P* value
	n = 16	n = 19	
Age, years: mean	51 (41–62)	53 (44–68)	0.72
Sex, females: number (%)	7 (44)	11 (57)	0.40
**Clinical features**			
Fever (%)		7 (37)	
Mononeuritis multiplex (%)		5 (26)	
Renal involvement (%)		4 (21)	
Arthritis (%)		2 (10)	
**Laboratory findings**			
White blood cell count, mean (/μL)		10,259 (5050–13,300)	
CRP, mean (mg/dL)		2.02 (0.18–3.14)	
Hemoglobin, mean (g/dL)		12.87 (11.8–14.2)	
Creatinine, mean (mg/dL)		0.78 (0.61–0.83)	
ANCA positivity (%)		2 (10)	

ANCA, anti-neutrophil cytoplasmic antibody; CRP, C-reactive protein.

Values in parentheses represent the percentage or interquartile range.

### Immune checkpoint molecule expression is elevated in the circulating T-cells in patients with vasculitis

**Figure 1:**
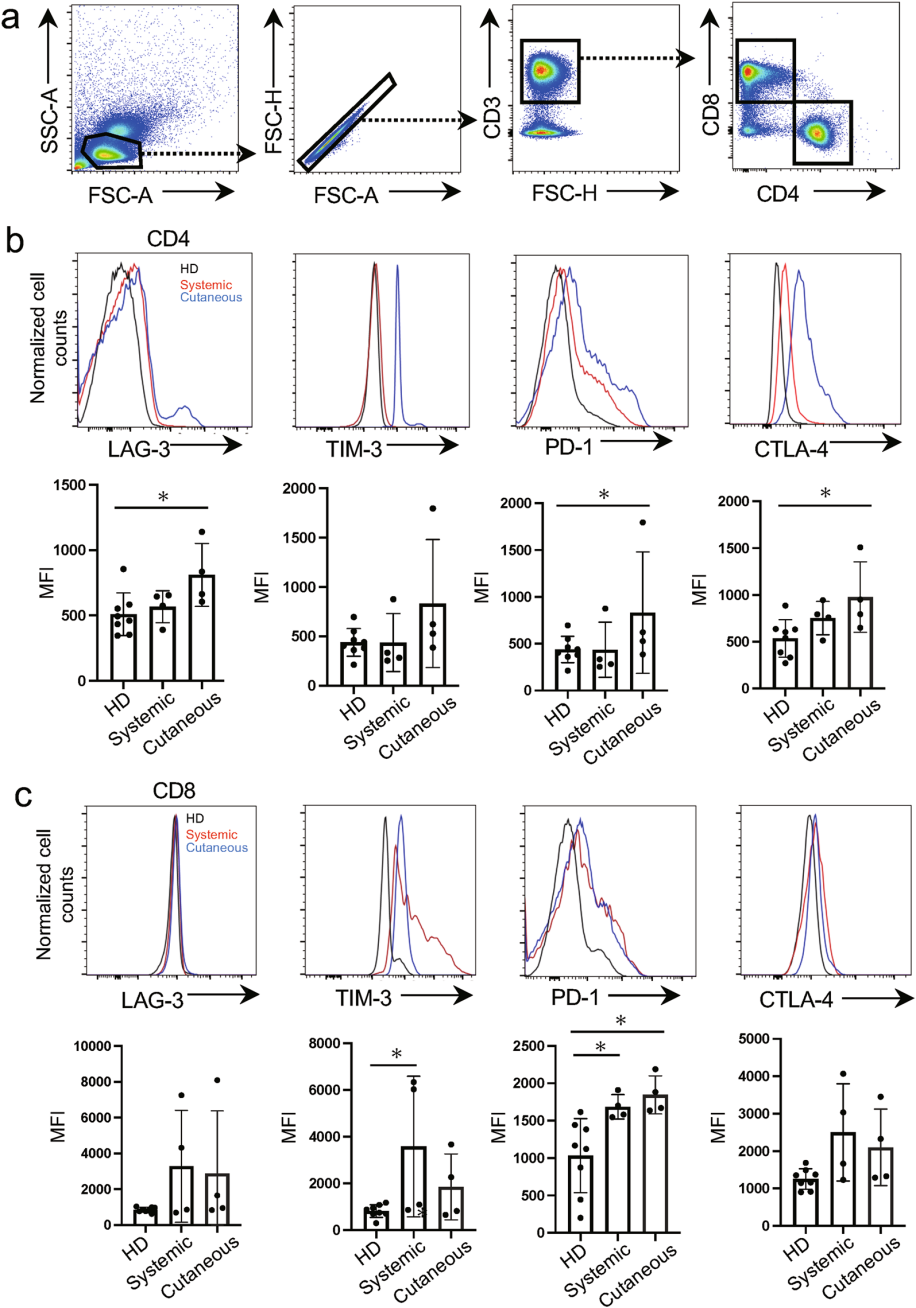
Immune checkpoint molecule expression levels are elevated in circulating T-cells derived from patients with vasculitis. (**a**) Representative gating strategy of T-cell subsets derived from peripheral blood mononuclear cells (PBMCs). (**b**) Representative histograms of immune checkpoint molecule expression in CD4^+^ T-cells isolated from PBMCs (red line: systemic vasculitis, blue line: cutaneous vasculitis, black line: healthy donors [HD]). Mean fluorescence intensity (MFI) of immune checkpoint molecule expression on CD4^+^ cells. (**c**) Representative histograms of immune checkpoint molecule expression in CD8^+^ T cells isolated from PBMC (red line: systemic vasculitis, blue line, cutaneous vasculitis, black line: HD). MFI of immune checkpoint molecule expression on CD8^+^ cells. Data indicate mean ± SEM. * *p* < 0.05.

We first analyzed the expression of immune checkpoint molecules on CD4^+^ and CD8^+^ T-cell populations in peripheral blood mononuclear cells (PBMC) based on the gating strategy shown in Fig. 1a. We observed that the mean fluorescence intensity (MFI) of PD-1 and CTLA-4 in the CD4^+^ T-cell population was notably increased in patients with cutaneous vasculitis compared to the HD (Fig. 1b). By contrast, the MFI of TIM3 and PD-1 in the CD8^+^ T-cell population was also markedly higher, especially in patients with systemic vasculitis compared to their HD counterparts (Fig. 1c). These findings suggest that immune checkpoint signaling is promoted in the circulating T-cells derived from patients with vasculitis.

### Frequencies of Ki67^+^PD-1^+^ and Ki67^+^IL-17^+^ cells are elevated in the circulating T-cells in patients with vasculitis

**Figure 2:**
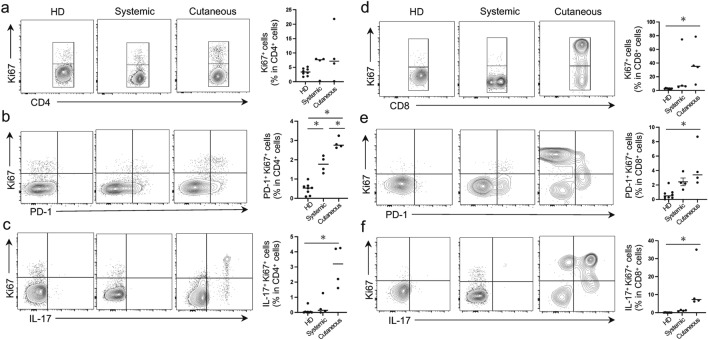
Frequencies of Ki67^+^ PD-1^+^ and Ki67^+^IL-17^+^ cells are elevated in the circulating T-cells in patients with vasculitis. (**a**–**c**) Representative flow contour plots and frequencies of (**a**) Ki67^+^ cells, (**b**) Ki67^+^PD-1^+^ cells, and (**c**) Ki67^+^IL-17^+^ cells within the CD4^+^ T-cell population in patients with systemic vasculitis, cutaneous vasculitis, and healthy donors (HDs). (**d**–**f**) Representative flow contour plots and frequencies of (**d**) Ki67^+^ cells, (**e**) Ki67^+^PD-1^+^ cells, and (**f**) Ki67^+^IL-17^+^ cells within the CD8^+^ T-cell population in patients with systemic vasculitis, cutaneous vasculitis, and HDs. Data indicate mean ± SEM. *p < 0.05.

T-cell proliferation is enhanced in various autoimmune diseases, including vasculitis^22^. Thus, we investigated the proliferative capacity of circulating T-cells in patients with vasculitis and HD by measuring the expression of Ki67, a marker of cellular replication. We observed that the percentage of Ki67^+^cells within the CD8^+^ cell population in patients with cutaneous vasculitis was higher than in HDs, while the percentage in the CD4^+^ cell population was not significantly different (Fig. 2a,d). This indicated that CD8^+^ T-cells were proliferating in the peripheral blood of patients with cutaneous vasculitis. Additionally, patients with cutaneous vasculitis demonstrated a markedly higher percentage of PD-1^+^ or interleukin (IL)-17^+^ cells within the Ki67^+^ T-cell population when compared to HDs (Fig. 2b, c, e, f). IL-17, produced by activated T helper 17 (Th17) cells, is an effector cytokine that induces vascular inflammation^23,24^. Therefore, T-cell proliferation, especially CD8^+^ cells, is promoted in cutaneous vasculitis and these proliferating T-cell populations includes both “suppressive” PD-1^+^ and “effector” IL-17^+^ cells.

### Expression levels of immune checkpoint molecules are altered in the skin of patients with cutaneous vasculitis

**Figure 3:**
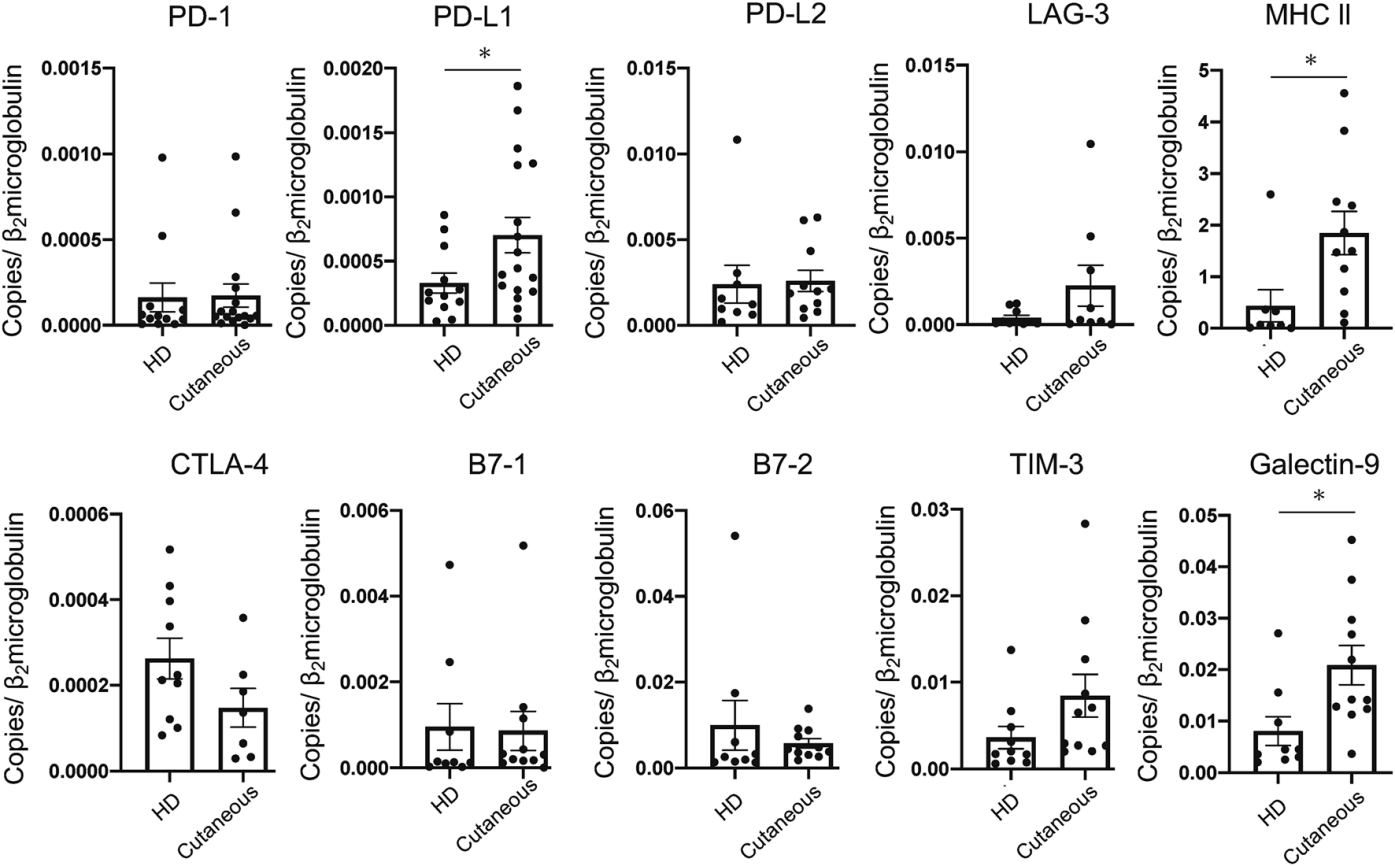
Expression levels of immune checkpoint molecules are altered in the skin in patients with cutaneous vasculitis. Real-time quantitative polymerase chain reaction analysis for immune checkpoint molecule levels in the RNA isolated from skin derived from patients with cutaneous vasculitis or HDs. Data indicate mean ± SEM. * *p* < 0.05.

It has been demonstrated that PD-1 expression is elevated in large arteries derived from patients with GCA, and the T-cells in GCA arteries preferentially express PD-1^21^. However, PD-1 expression in small- to medium-sized vessels with cutaneous vasculitis is currently unclear. Moreover, other checkpoint molecules expressed in the cutaneous vessels of the skin have not yet been studied. To further understand the immune checkpoint molecule signatures in the skin, we analyzed mRNA levels of checkpoint receptors and their ligands in the affected skin and the healthy skin obtained by skin biopsies. The inhibitory immune checkpoint molecules studied in this study were described in Supplemental Table 1. We found that the messenger RNA levels of PD-L1, major histocompatibility complex (MHC) class II, and Galectin-9, ligands for PD-1, LAG-3, and TIM-3, respectively, were elevated in the affected skin (Fig. 3). These results suggested that in the inflamed skin, although the immune checkpoint receptor expression was not altered, some of the immune checkpoint receptor ligands were highly expressed and playing roles in the local inflammatory environments.

### Analysis of programmed death-ligand 1 expression in the skin derived from patients with cutaneous vasculitis

**Figure 4:**
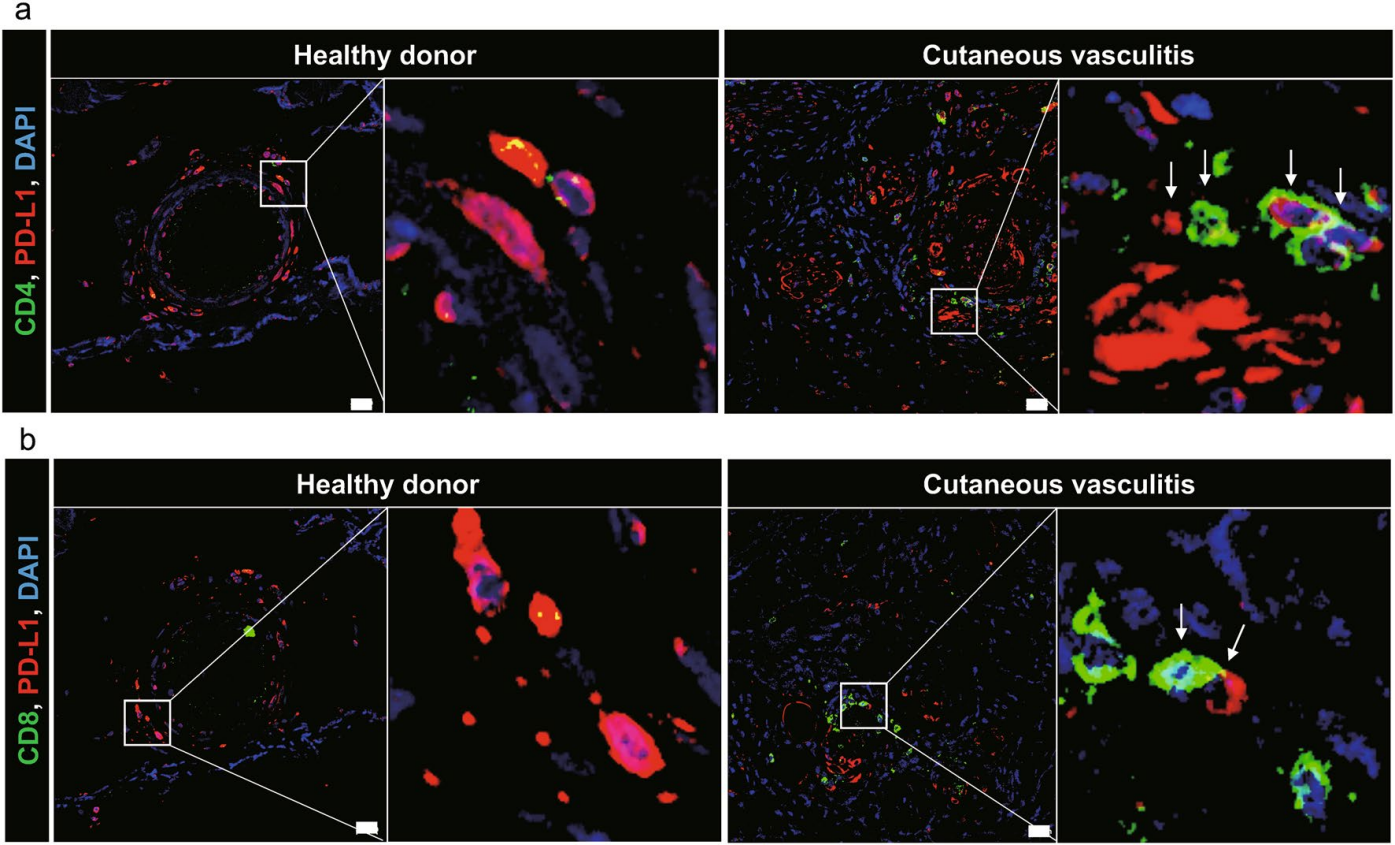
Analysis of PD-L1 expression in the skin derived from patients with cutaneous vasculitis (**a, b**) Skin was collected from patients with cutaneous vasculitis or healthy donors and stained with anti-CD4 (green, **a**), anti-CD8 (green, **b**), and anti-PD-L1 (red). Insets indicate the magnified images of the vessel wall. Arrows show T-cells adjacent to the PD-L1 protein. Scale bars = 20 µm.

PD-1 and its ligands are the most studied immune checkpoint targets that regulate T-cell activation, tolerance, and immune-mediated organ damage^5^. Moreover, the blockade of PD-1 or its ligand PD-L1 has been successful in treating a wide variety of cancers. Furthermore, PD-L1 can also be used as a predictive biomarker for clinical response to PD-1 signal inhibitors^25^. Additionally, growing evidence demonstrates that the impaired PD-L1/PD-1 pathway plays an important role in a variety of different autoimmune diseases^26^. Thus, we focused on PD-L1 and histologically analyzed the skin tissue of patients with cutaneous vasculitis to visualize the location of PD-L1. We identified CD4^+^ (Fig. 4a) and CD8^+^ (Fig. 4b) T-cells, as well as the PD-L1 protein expression around the inflamed vessels, however, there were very few T-cells and little PD-L1 expression in HDs. Although CD4^+^ or CD8^+^ T-cells did not express PD-L1, these T-cells were localized adjacent to the vessels expressing PD-L1. These results demonstrate that PD-L1 is overexpressed in the affected skin, and we speculate that T-cells infiltrating the skin vessels may be receiving inhibitory signals from PD-L1 expressed in the vessels.

### Skin T-cells derived from patients with cutaneous vasculitis are proliferative and express Granzyme B

**Figure 5:**
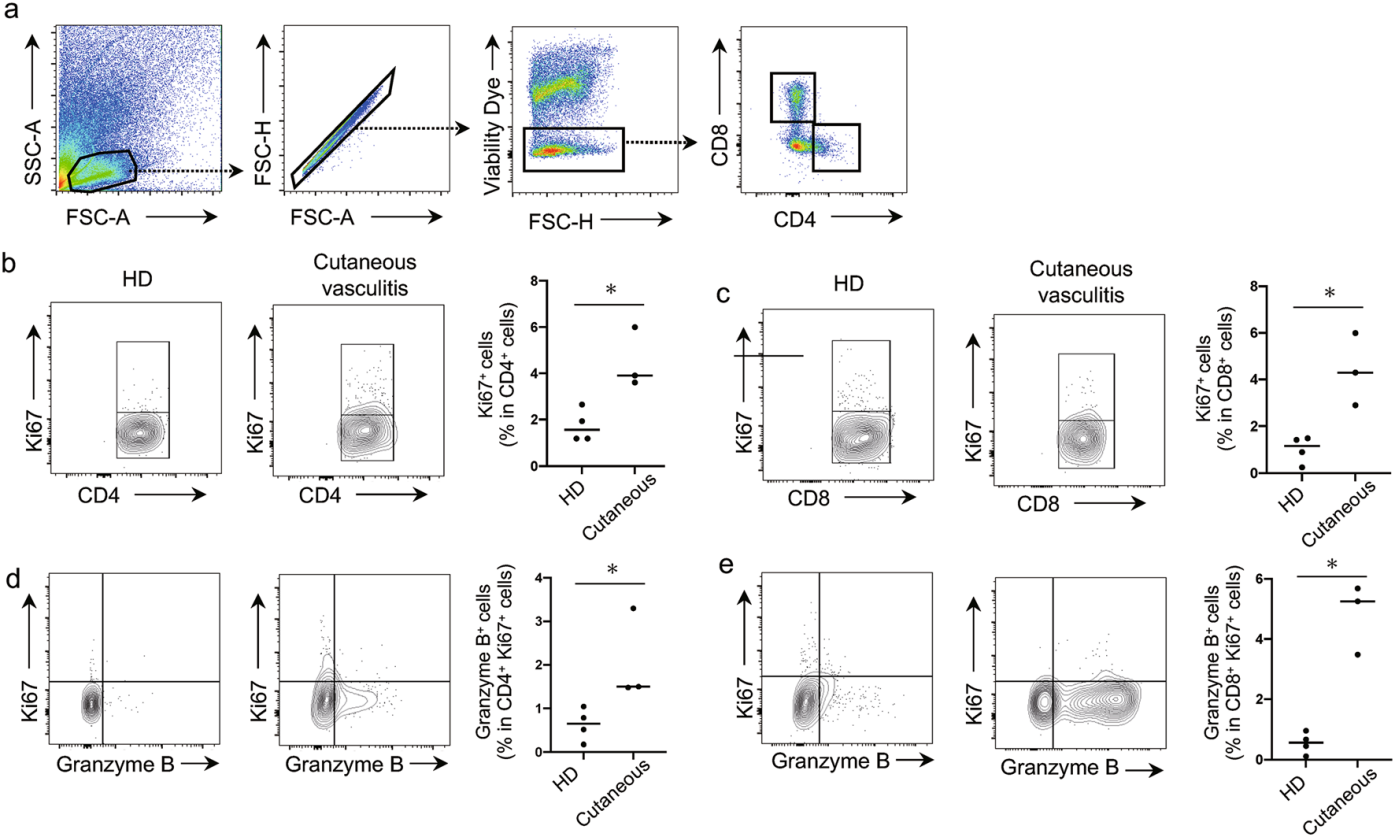
Skin T-cells derived from patients with cutaneous vasculitis proliferate and express Granzyme B. (**a**) Representative gating strategy of T-cell subsets derived from the skin. (**b**, **c**) Representative flow contour plots and frequencies of Ki67^+^cells within (**b**) CD4^+^ or (**c**) CD8^+^ T-cell populations in patients with cutaneous vasculitis and healthy donors (HD). (**d**, **e**) Representative flow contour plots and frequencies of Ki67^+^ Granzyme B^+^ cells within (**d**) CD4^+^ or (**e**) CD8^+^ T-cell populations in patients with cutaneous vasculitis and HD. Data indicate mean ± SEM. *p < 0.05.

To further characterize the T-cell phenotype in the inflamed skin of patients with cutaneous vasculitis, cell suspensions were obtained after enzyme digestion and subsequently analyzed by flow cytometry. CD4^+^ and CD8^+^ T-cells were determined after gating on live cells (Fig. 5a). We observed that the population of Ki67^+^cells in both CD4^+^ and CD8^+^ cell populations in the inflamed skin was higher than in those derived from HDs (Fig. 5b, c). Additionally, there was a markedly higher percentage of Granzyme B^+^ T-cells in the Ki67^+^ T-cell populations derived from the skin of patients with cutaneous vasculitis compared with HDs (Fig. 5d, e). However, PD-1 expression in the skin-derived T-cells was not significantly different between HDs and patients with cutaneous vasculitis (data not shown).

### Plasma soluble programmed death-ligand 1 is increased in patients with systemic vasculitis and correlates with C-reactive protein levels

**Figure 6:**
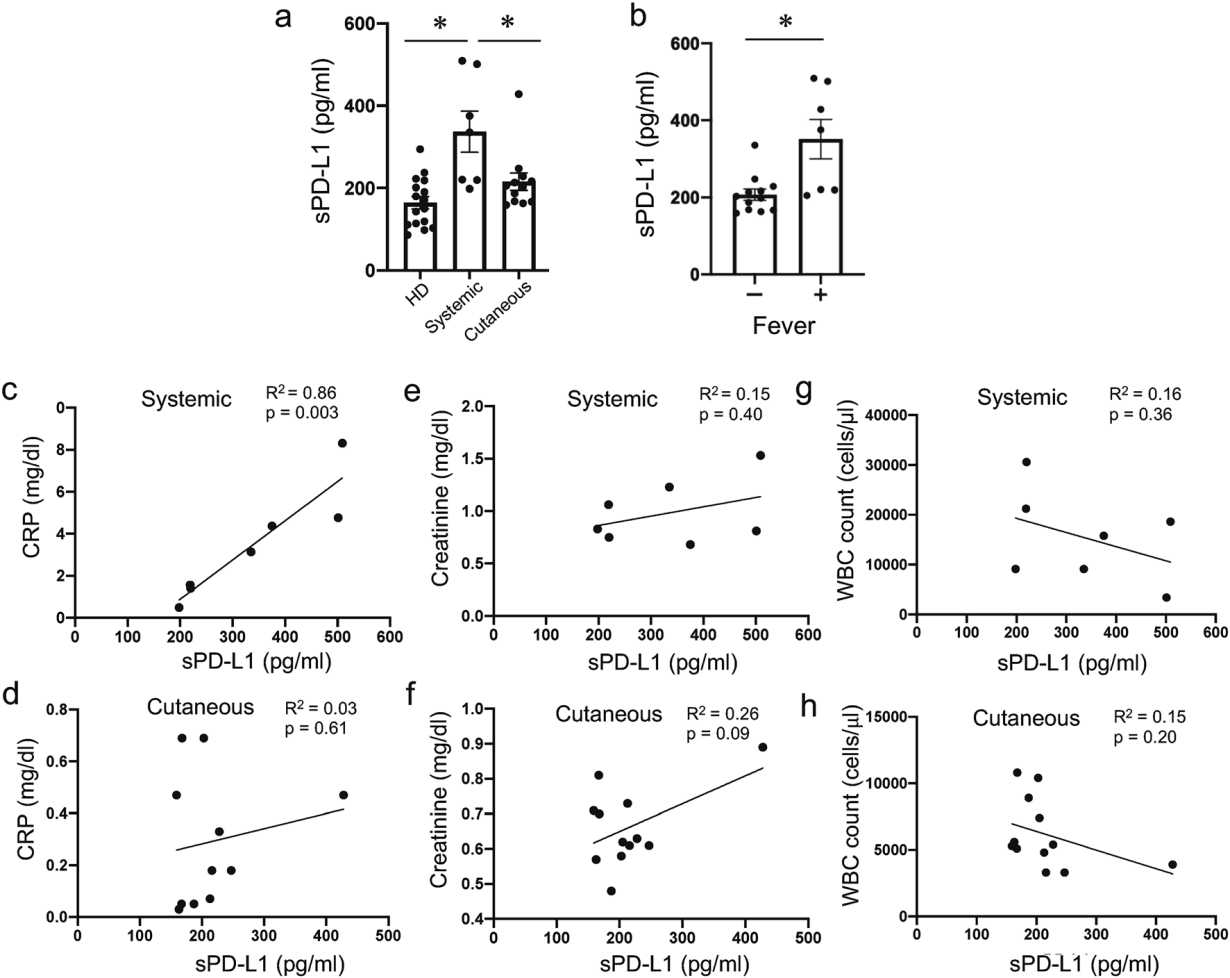
Plasma soluble PD-L1 in patients with vasculitis and correlation with CRP, plasma creatinine levels, and WBC count. (**a**) Plasma soluble PD-L1 levels in patients with systemic vasculitis, cutaneous vasculitis, and healthy donors (HDs). Data indicate mean ± SEM. * *p* < 0.05. (**b**) Plasma soluble PD-L1 levels in patients with fever or without fever. Data indicate mean ± SEM. *p < 0.05. (**c**–**h**) Correlation between CRP, white blood cell count, and creatinine levels in the plasma alongside plasma soluble PD-L1 levels.

Previous reports have shown that blood sPD-L1 levels are increased and associated with disease activities in various autoimmune diseases, including rheumatoid arthritis, systemic lupus erythematosus, autoimmune hepatitis, systemic sclerosis, and immune thrombocytopenia^27–31^. To determine if plasma sPD-L1 levels have an association with other clinical parameters in vasculitis, we examined the levels of plasma sPD-L1 in patients with vasculitis. Plasma sPD-L1 levels were notably elevated in patients with systemic vasculitis compared to cutaneous vasculitis or HDs (Fig. 6a). Among patients with vasculitis, a group that presented with fever (above 37.5 °C) showed elevated plasma sPD-L1 levels (Fig. 6b). Plasma sPD-L1 levels were positively correlated with plasma C-reactive protein (CRP) in both systemic and cutaneous vasculitis (Fig. 6c,d). Furthermore, plasma sPD-L1 levels were weakly correlated with plasma creatinine levels in cutaneous vasculitis (Fig. 6f). Contrastingly, there was no significant correlation between sPD-L1 levels and the creatinine levels in systemic vasculitis, and the number of white blood cells in the peripheral blood in both systemic and cutaneous vasculitis (Fig. 6e, g, h).

## Discussion

The clinical symptoms of vasculitis are variable and can affect any organ, including the kidneys, lungs, nerves, gastrointestinal tract, and skin^32^. The skin is a privileged organ in the setting of vasculitis since it is easily accessible for physical examinations and safe biopsies when compared to other organs. Therefore, it is suitable for the characterization of immune cell features^33^. In this study, we sought to characterize the immune checkpoint signatures in both the inflamed skin and peripheral blood of patients with vasculitis and HDs.

We demonstrated that T-cells in the peripheral blood of patients with cutaneous vasculitis preferentially expressed inhibitory immune checkpoint receptors, such as PD-1. In patients with cutaneous vasculitis, circulating CD8^+^ T-cells were proliferating, including both IL-17 and PD-1 expressing cells. This suggested that T-cells with effector and suppressive phenotypes were co-existing in the peripheral blood. In the affected skin, ligands of inhibitory immune checkpoints such as PD-L1, MHC class II, and Galectin-9 were highly expressed, although the expression of their receptors was not altered. Histologically, PD-L1 was also highly expressed in the vessels of the dermis in patients with cutaneous vasculitis, and CD4^+^ and CD8^+^ T-cells were located adjacent to the PD-L1-expressing vessels. By contrast, both CD4^+^ and CD8^+^ T-cells were proliferating in the affected skin, with both containing cytotoxic granzyme B. In addition, our data have demonstrated that plasma sPD-L1 was positively correlated with CRP levels in patients with systemic vasculitis.

T-cell exhaustion is a state of T-cell dysfunction that arises during many chronic infections and cancers induced by persistent antigen exposure and/or inflammation^34^. It is characterized by hierarchical loss of effector functions, sustained upregulation of multiple inhibitory receptors, altered expression and use of key transcription factors, metabolic derangements, and a failure to transition to quiescence, acquiring antigen-independent memory T-cell homeostatic responsiveness^35^. Higher levels of PD-1 expression are often considered indicative of an exhausted state. Moreover, previous studies show that the PD-L1/PD-1 pathway regulates the induction and maintenance of peripheral tolerance while simultaneously protecting tissues from autoimmunity^36^. Indeed, PD-1-deficient mice develop lupus-like glomerulonephritis, arthritis, and cardiomyopathy through the generation of autoantibodies^37^. Here, we demonstrate that circulating T-cells in patients with vasculitis showed exhausted phenotype, characterized by the elevated expression of multiple inhibitory immune checkpoint receptors. However, in the affected skin, T-cells maintained their cytotoxic function by expressing granzyme B, and thus, they are not fully exhausted. Our results suggested that immune checkpoint molecules might be differentially modulated in the skin and peripheral blood in patients with vasculitis.

Expression of PD-L1 is observed in hematopoietic cells, including T cells, B cells, macrophages, dendritic cells, and non-hematopoietic healthy tissue cells, including vascular endothelial cells, keratinocytes, pancreatic islet cells, and astrocytes^38^. PD-L1 expression can be induced or maintained by many inflammatory cytokines, including interferons, tumor necrosis factor (TNF)-α, IL-6, and IL-17^38^. In the present study, PD-L1 was elevated in the plasma of patients with systemic vasculitis, whereas it was not significantly altered in patients with cutaneous vasculitis. In addition, plasma PD-L1 levels were strongly associated with CRP titer in patients with systemic vasculitis. Although PD-L1-producing cells have not been identified, it is speculated that PD-L1 was induced by inflammatory signals in the microenvironment, such as IL-17 produced by circulating T-cells. IL-6 is the major cytokine that induces transcription of CRP during the acute phase response^39^, as well as one of the fever-inducible cytokines^40^. The elevation of plasma sPD-L1 and CRP levels and high fever, may be mediated by inflammatory cytokines present either in the blood or skin. Therefore, the inflammatory milieu in vasculitis presumably leads to increased sPD-L1 production which compensates for the aberrant T-cell activation and maintains homeostasis.

Consistent with our data, the frequency of PD-1^+^ T-cells in the peripheral blood of patients with GPA was elevated^20^. However, the frequency of PD-1^+^ T-cells in the peripheral blood of patients with GCA was decreased^21^. In the previous two reports, the patient cohorts included both active and quiescent disease. Moreover, most of the patients were already being treated with corticosteroids or immunosuppressive agents. In contrast, our patient cohort consists of newly diagnosed, active disease patients without immunosuppressive therapies. Thus, it is possible that different disease status and medications affect PD-1 expression in T-cells. Alternatively, different disease mechanisms according to the size of affected vessels might explain the discrepancies between the results.

Some limitations in the present study need to be considered. First, we focused mainly on the PD-L1/PD-1 pathway, but have not fully evaluated other immune checkpoint molecules, including CTLA-4 and TIM3. Considering the complexity of multiple inhibitory molecules in the immune system, it is speculated that in addition to PD-L1/PD-1, other inhibitory molecules may also regulate inflammation in vasculitis. Second, the small sample size could have influenced the results. Third, we have not assessed the correlation between immune checkpoint profiles and disease prognosis in this study. Further studies will be required to clarify the immune checkpoint molecule mechanisms at work, as well as the correlation these molecules have with the disease prognosis.

## Materials and methods

### Study population

Blood and skin tissue were obtained from patients with vasculitis who visited us between 2018 and 2020. These participants were divided into patients with a diagnosis of cutaneous arteritis (CA, n = 5), IgA vasculitis (n = 9), eosinophilic granulomatosis with polyangiitis (EGPA, n = 3), microscopic polyangiitis (MPA, n = 1), and other AAVs (n = 1). CA and IgA vasculitis were categorized as cutaneous vasculitis, while EGPA, MPA, and AAVs were categorized as systemic vasculitis.

HD without systemic diseases undergoing surgery were selected as controls, whose normal skins around the surgical margin were taken for the study.

Clinical information on the patients and HDs is provided in Table 1. Patients with vasculitis were diagnosed according to the American College of Rheumatology criteria^41^ and the Watts algorithm^42^. The experimental protocol was approved by the Ethics Committee of the Tohoku Medical and Pharmaceutical University. Written informed consent was obtained from all the participants. All methods were performed in accordance with the relevant guidelines and regulations and with the Helsinki Declaration.

### Cell isolation

Plasma and PBMCs were isolated from whole blood in CPT tubes (Becton Dickinson, BD) according to the manufacturer's instructions. Dermal tissue was minced and then incubated overnight at 37 °C in RPMI containing 1 mg/mL collagenase P (Roche) and 0.02 mg/ml DNAse (Sigma-Aldrich) as described previously^43^. A cell suspension was obtained by mashing the tissue through a 70 µm strainer.

### Flow cytometry

Single cells were incubated with Human TruStain FcX (BioLegend) to block Fc receptors. These were then stained with Ghost Dye™ Red 780 Viability Dye (Cell Signaling Technology) to identify dead cells and the following fluorochrome-conjugated anti-mouse monoclonal antibodies (mAbs): CD103-PerCP/Cy5.5 (Ber-ACT8), CTLA-4- PerCP/Cy5.5 (BNI3), Granzyme B-FITC (QA16A02), PD-1-PE (EH12.2H7), CD27- PE/Cy7 (LG.3A10), CD3- PE/Cy7 (HIT3a), CD8a-AF700 (RPA-T8), CD4-APC (OKT4), CD127-BV711 (A019D5), Ki-67-BV605 (Ki-67), CD45RA-BV605 (HI100), CCR7-BV510 (G043H7), CD69-BV421 (FN50), and IFNγ-BV421 (4S.B3) (all from BioLegend). Data were acquired on a LSRFortessa X-20 (BD) and analyzed with FlowJo software.

### Quantitative real-time polymerase chain reaction

Skin tissue was isolated and homogenized in Trizol (Thermo Fisher Scientific) and RNA was isolated according to the manufacturer’s instruction. Purified RNA was then converted to cDNA by reverse transcription (TaqMan Reverse Transcriptase Reagents, Thermo Fisher Scientific). Quantitative polymerase chain reactions (qPCR) in the presence of SYBR Green (PowerUp SYBR Green Master Mix, Applied biosystems) were performed on a QuantStudio 3 Instrument (Thermo Fisher Scientific) and normalized to beta 2-microglobulin using the following primers: *Pd-I,* 5′–CCA GGA TGG TTC TTA GAC TCC C–3′ (forward) and 5′–TTT AGC ACG AAG CTC TCC GAT–3′ (reverse); *Pd-l1*, 5′–TGG CAT TTG CTG AAC GCA TTT–3′ (forward) and 5′–TGC AGC CAG GTC TAA TTG TTT T–3′ (reverse); *Pdl-2,* 5′–ACC GTG AAA GAG CCA CTT TG–3′ (forward) and 5′-GCG ACC CCA TAG ATG ATT ATG C-3′ (reverse); *Ctla-4*, 5′-GCC CTG CAC TCT CCT GTT TTT-3′ (forward) and 5′-GGT TGC CGC ACA GAC TTC A-3′ (reverse); *B7-1,* 5′-AAA CTC GCA TCT ACT GGC AAA-3′ (forward) and 5′-GGT TCT TGT ACT CGG GCC ATA-3′ (reverse); *B7-2,* 5′-CTG CTC ATC TAT ACA CGG TTA CC-3′ (forward) and 5′-GGA AAC GTC GTA CAG TTC TGT G-3′ (reverse); *Tim-3,* 5′-CTG CTG CTA CTA CTT ACA AGG TC-3′ (forward) and 5′-GCA GGG CAG ATA GGC ATT CT-3′ (reverse); *Lag3,* 5′-GCG GGG ACT TCT CGC TAT G-3′ (forward) and 5′-GGC TCT GAG AGA TCC TGG GG-3′ (reverse); *MhcII,* 5′-GAG CAG GTT AAA CAT GAG TGT CA-3′ (forward) and 5′-CTC TCC ACA ACC CCG TAG T-3′ (reverse); and *Galectin-9,* 5′-GGA CGG ACT TCA GAT CAC TGT-3′ (forward) and 5′-CCA TCT TCA AAC CGA GGG TTG-3′ (reverse).

### Immunofluorescence microscopy

Paraffin-embedded skin tissues were deparaffinized and then autoclaved for 5 min at 121 °C in 10 mM Tris Buffer in 1 mM EDTA. They were then removed from the heat and kept at 25 °C for 20 min. Sections were blocked with protein block (DakoCytomation) for 15 min and stained with mouse anti-PD-L1 antibody (Abcam), rabbit anti-CD4 antibody (Diagnostic BioSystems), and rabbit anti-CD8 antibody (Abcam) overnight at 4 °C. Alexa Fluor 488–conjugated donkey anti–rabbit IgG antibody (Abcam) or Alexa Fluor 568–conjugated donkey anti–mouse IgG antibody (Abcam) was used as a secondary antibody and samples were incubated with these for 30 min at 25 °C. The slides were examined using a fluorescence microscope (Carl Zeiss).

### Detection of plasma soluble programmed death-ligand 1 levels

Plasma sPD-L1 levels in patients with vasculitis and HDs were measured by chemiluminescence enzyme immunoassays, as previously described^44^.

### Statistical analysis

All statistical analyses were performed in Prism 8 (GraphPad Software). Data are expressed as the mean ± SEM. Significance for the differences between groups was determined using unpaired two-tailed Student’s *t*-test. *P* value in multiple groups was calculated using ordinary one-way analysis of variance (ANOVA) with a post hoc Tukey’s test for multiple comparisons. Spearman's correlation coefficient was used to detect the correlation between different study parameters. *P* value less than 0.05 was considered statistically significant.

## Supplementary Information

Supplementary Information.

## Data Availability

No datasets were generated during the current study.

## Abbreviations

PD-1: Programmed cell death 1
PD-L1: Programmed death-ligand 1
CRP: C-reactive protein
CTLA-4: Cytotoxic T lymphocyte-associated molecule-4
LAG-3: Lymphocyte activation gene-3
TIM-3: T cell immunoglobulin and mucin-domain containing-3
TIGHT: T cell immunoglobulin and ITIM domain
VISTA: V-domain Ig suppressor of T cell activation
irAEs: Immune-related adverse events
ANCA: Anti-neutrophil cytoplasmic antibody
AAV: ANCA-associated vasculitis
GCA: Giant cell arteritis
GPA: Granulomatosis with polyangiitis
sPD-L1: Soluble PD-L1
EGPA: Granulomatosis with polyangiitis
MPA: Microscopic polyangiitis
HD: Healthy donors
PBMC: Peripheral blood mononuclear cells
MFI: Mean fluorescence intensity
IL: Interleukin
MHC: Major histocompatibility complex
TNF: Tumor necrosis factor
