# Supplementary material for: Immune checkpoint molecule expression is altered in the skin and peripheral blood in vasculitis
Source: Sci Rep. 2021 Oct 8;11:20019. doi: 10.1038/s41598-021-99558-5 (PMC8501116; doi:10.1038/s41598-021-99558-5)
Supplement: Supplementary file 1 — Supplementary Information. [file 41598_2021_99558_MOESM1_ESM.docx]

**SUPPLEMENTAL INFORMATION**

**Table S1. List of inhibitory immune checkpoint molecules**

| **Receptor** | **Ligand** |
| --- | --- |
| PD-1 (programmed cell death 1 receptor) | PD-L1 (CD274), PD-L2 (CD273) |
| CTLA-4 (cytotoxic T lymphocyte-associated molecule-4) | B7-1 (CD80), B7-2 (CD86) |
| LAG-3 (lymphocyte activation gene-3) | MHC class ll |
| TIM-3 (T cell immunoglobulin and mucin-domain containing-3) | Galectin-9 |
